# Elranatamab in Japanese patients with relapsed/refractory multiple myeloma: results from MagnetisMM-2 and MagnetisMM-3

**DOI:** 10.1093/jjco/hyae068

**Published:** 2024-05-24

**Authors:** Shinsuke Iida, Satoshi Ito, Hisayuki Yokoyama, Tadao Ishida, Yuya Nagai, Hiroshi Handa, Shigeki Ito, Yoichi Kamei, Masatoshi Nakamura, Kenshi Suzuki

**Affiliations:** Department of Hematology and Oncology, Nagoya City University Graduate School of Medical Sciences, Kawasumi Mizuho-cho, Mizuho-ku, Nagoya, Aichi 467-8602, Japan; Department of Internal Medicine III, Division of Hematology and Cell Therapy, Yamagata University Graduate School of Medicine, Yamagata, Japan; Department of Hematology and Rheumatology, Tohoku University Graduate School of Medicine, Sendai, Japan; Department of Hematology, Japanese Red Cross Medical Center, Tokyo, Japan; Department of Hematology, Kobe City Medical Center General Hospital, Kobe, Japan; Department of Hematology, Gunma University Graduate School of Medicine, Maebashi, Japan; Division of Hematology & Oncology, Department of Internal Medicine, School of Medicine, Iwate Medical University, Yahaba, Japan; Pfizer R&D Japan GK, Tokyo, Japan; Pfizer R&D Japan GK, Tokyo, Japan; Department of Hematology, Japanese Red Cross Medical Center, Tokyo, Japan

**Keywords:** multiple myeloma, bispecific antibodies, B-cell maturation antigen, Japanese patients, triple-class refractory

## Abstract

**Background:**

Despite advances, most patients with multiple myeloma (MM) experience relapse and repeat multiple treatment lines, highlighting an unmet need for patients with relapsed or refractory MM (RRMM). Bispecific antibodies are a new option, but their efficacy and safety in Japanese patients are unknown.

**Methods:**

This was an analysis of Japanese patients receiving elranatamab monotherapy in MagnetisMM-2 (NCT04798586) and MagnetisMM-3 (NCT04649359). Both studies evaluated a priming dose regimen of elranatamab followed by weekly subcutaneous doses, in patients with disease progression while receiving or who were intolerant to ≥3 prior therapies (≥1 proteasome inhibitor, ≥1 immunomodulatory drug and ≥1 anti-CD38 monoclonal antibody). The primary endpoints were dose limiting toxicities (DLTs) in MagnetisMM-2 and confirmed objective response rate (ORR) in MagnetisMM-3. In both, key secondary endpoints included safety, tolerability, duration of response, time to response, progression-free survival and overall survival.

**Results:**

In MagnetisMM-2 (*N* = 4) and MagnetisMM-3 (*n* = 12), median ages were 68.5 and 66.5 years, respectively. No DLTs were observed in MagnetisMM-2. ORRs were 50.0% (95% CI, 6.8–93.2) and 58.3% (95% CI, 27.7–84.8) in MagnetisMM-2 and MagnetisMM-3, respectively. All patients experienced treatment-emergent adverse events in MagnetisMM-2 (grade 3/4: 75.0%) and MagnetisMM-3 (grade 3/4: 100%); cytokine release syndrome occurred in 100% (grade 3/4: 25.0%) and 58.3% (no grade 3/4) of patients, respectively. Neither study reported immune effector cell–associated neurotoxicity syndrome.

**Conclusions:**

No new safety signals were observed, and ORRs were similar to that of the overall MagnetisMM-3 trial population, supporting further studies of elranatamab in Japanese patients with RRMM.

**ClinicalTrials.gov identifier:** NCT04798586 (MagnetisMM-2), NCT04649359 (MagnetisMM-3).

## Teaser

Elranatamab monotherapy demonstrated clinical efficacy and manageable safety in Japanese patients with relapsed or refractory multiple myeloma, similar to the overall patient population and supporting further studies in Asian populations.

## Introduction

Multiple myeloma (MM) is an incurable plasma cell neoplasm characterized by end-organ damage and a range of debilitating symptoms including, but not limited to, lytic bone lesions, anaemia, hypercalcemia and renal impairment (1). Japan has the highest crude incidence of MM in Asia due to its aging population; the number of diagnosed cases has steadily increased since 1975 with ≈7600 new cases reported in 2019 (2–6). The 5-year overall survival (OS) rate for patients with MM is ≈50%, and real-world data of Japanese patients with MM have shown that the 5-year OS rate is ≈46% (7,8).

The MM treatment landscape has expanded rapidly over the last 20 years to provide more antimyeloma therapies with different mechanisms of action for patients both in Japan and globally (2,9). However, the integration of these therapies into an optimal treatment sequence to maximize clinical benefit in later lines of treatment, particularly after triple-class exposure (immunomodulatory drugs [IMiDs], proteasome inhibitors [PIs] and anti-CD38 monoclonal antibodies [mAbs]), has become increasingly challenging (9). In addition, despite these therapeutic advances in MM, only a limited number of patients achieve long-term disease control, and the majority of patients eventually experience relapse and receive multiple lines of treatment (8,10). Therefore, an unmet need exists for treatments for MM resistant to prior therapies (8).

More recently, B-cell maturation antigen (BCMA) has emerged as a target because its expression is a hallmark of myeloma cells (8,9,11–13). Elranatamab is a humanized bispecific antibody targeting BCMA on myeloma cells and CD3 on T cells (8,14). Elranatamab induces a cytotoxic T-cell response against myeloma cells (14).

In the ongoing phase 1 MagnetisMM-1 study (NCT03269136) in patients with relapsed or refractory MM (RRMM), elranatamab had a manageable safety profile when given subcutaneously (SC) weekly or every 2 weeks at doses from 215 to 1000 μg/kg. Patients receiving the recommended phase 2 dose of 1000 μg/kg (or equivalent 76-mg fixed dose) also received a 600-μg/kg priming dose (or equivalent 44-mg fixed dose) (15). The reported objective response rate (ORR) was 64%, with 38% of patients achieving complete response (CR) or better (15). Preliminary data from the ongoing MagnetisMM-3 study (NCT04649359) showed that elranatamab was well tolerated, with an ORR of 61% in patients naive to prior BCMA-directed therapies (16).

Although these data are promising, the trial populations have been primarily based in the USA and Europe. Some treatments for RRMM, such as thalidomide and bortezomib, have shown differences in safety profiles between Western populations and Japanese or other Asian populations (17–20). In Korea and Taiwan, there was a lower frequency of thromboembolic events (3.9% and 3.5%, respectively) than the worldwide incidence (10% ~ 58%) for patients receiving thalidomide treatment without TE prophylaxis (17,18). The incidence of pulmonary complications associated with bortezomib treatment was shown to be higher in Japan (4.5% with a risk minimization action plan) compared to the worldwide incidence (0.08%) (20). Other MM therapies, such as the ADC belantamab mafodotin and the triplet regimens ixazomib-lenalidomide-dexamethasone and isatuximab-carfilzomib-dexamethasone, have shown similar safety profiles in Asian and overall trial populations (21–23). Chimeric antigen receptor (CAR) T-cell therapies ciltacabtagene autoleucel and idecabtagene vicleucel have also been tested in Japanese patients, with results similar to the overall trial populations (24,25). To date, there are no published data on comparisons of safety profiles for bispecific antibodies approved for MM treatment. Given these highlighted examples of differences between Asian and Western/global populations in the safety of various MM therapies, it is important to ascertain the efficacy and safety of elranatamab in an Asian population.

This analysis was conducted to assess data in Japanese patients with RRMM from the open-label, phase 1, MagnetisMM-2 (NCT04798586) study and the phase 2 MagnetisMM-3 study.

## Patients and methods 

### Study design and patient population

#### MagnetisMM-2

MagnetisMM-2 was a non-randomized, open-label, multicentre, phase 1 study to evaluate the safety, immunogenicity, pharmacokinetics (PK) and pharmacodynamics of elranatamab monotherapy in Japanese patients with RRMM.

Eligible patients were age ≥ 20 years with RRMM and measurable disease, as defined by International Myeloma Working Group (IMWG) criteria, with an Eastern Cooperative Oncology Group performance status (ECOG PS) of 0–2 (26). Patients were required to have experienced disease progression on or be intolerant of ≥3 prior therapies, including a PI, an IMiD and an anti-CD38 mAb. Adequate bone marrow, renal and liver function was also required to be deemed eligible (see Supplementary Methods).

The study, opened only in Japan, started in March 2021, and this manuscript reports results as of the data cutoff of 27 May 2022.

#### MagnetisMM-3

The ongoing MagnetisMM-3 trial is a non-randomized, open-label, multicenter, phase 2 registrational study to evaluate the efficacy and safety of elranatamab monotherapy in patients with RRMM (including those from Japan) naive to prior BCMA-directed therapy (Cohort A) and patients who received prior BCMA-directed antibody–drug conjugate or BCMA-directed chimeric antigen receptor T-cell therapy (Cohort B).

Eligible patients were age ≥18 years with RRMM and measurable disease, as defined by IMWG criteria, with an ECOG PS of 0–2 (26). Patients were required to be refractory to ≥1 PI, ≥1 IMiD and ≥1 anti-CD38 mAb and had experienced relapse or had disease refractory to their last anti-MM regimen. Patients also had adequate bone marrow, renal and liver function (see Supplementary Methods).

In the present analysis, results are presented for Cohort A of this ongoing study, which started in February 2021, and are from the 14 October 2022 data cutoff.

#### Study oversight

Both MagnetisMM-2 and MagnetisMM-3 were conducted in accordance with the ethics principles of the Declaration of Helsinki and the Council for International Organizations of Medical Sciences International Ethical Guidelines, and the International Council for Harmonisation Good Clinical Practice Guidelines. All relevant documents including amendments were reviewed and approved by the institutional review board and/or ethics committee of each participating center. All patients provided written informed consent.

### Treatment and dosing schedule

In MagnetisMM-2, an initial dose of elranatamab was administered as a single priming dose of 600 μg/kg SC 7 days prior to Day 1 of the first cycle, followed by 1000 μg/kg SC weekly in 3-week cycles. Premedications were given 60 minutes prior to the priming regimen and the first full dose of elranatamab and included acetaminophen 650 mg, diphenhydramine 25 mg and dexamethasone 20 mg. Similar premedications were given prior to other elranatamab doses at the discretion of the investigator.

In MagnetisMM-3, SC elranatamab was administered weekly with step-up priming doses of 12 mg on Day 1 and 32 mg on Day 4, followed by a full dose of 76 mg weekly (starting on Day 8). The same premedications were given as in MagnetisMM-2. If a patient received ≥6 cycles and achieved partial response or better with responses persisting for ≥2 months, the dose interval was changed to once every 2 weeks (dose intervals were to return weekly if disease burden increased).

### Outcomes

#### MagnetisMM-2

The primary outcome in MagnetisMM-2 was dose-limiting toxicities (DLTs) in the first 4 weeks of treatment, defined as febrile neutropenia (absolute neutrophil count <1000/mm^3^ with a single temperature of ≥38°C for >1 hour), grade 4 neutropenia lasting >7 days, grade ≥3 neutropenia with infection, grade 4 thrombocytopenia, grade 3 thrombocytopenia with grade ≥2 bleeding, grade 4 adverse events (AEs), grade 3 AEs lasting ≥5 days despite optimal supportive care, grade 3 cytokine release syndrome (CRS; except those CRS events that were not maximally treated or improved to grade ≤1 within 48 hours), grade 4 CRS, confirmed drug-induced liver injury, or grade 4 laboratory abnormalities deemed clinically significant by the investigator. Clinically important or persistent toxicities not included in the above definition could also be considered DLTs following investigator and sponsor review.

Key secondary endpoints for MagnetisMM-2 included assessments of safety and tolerability. Treatment-emergent AE (TEAE) severity was graded by National Cancer Institute Common Terminology Criteria for Adverse Events version 5.0 (NCI CTCAE v5.0), except for CRS and immune effector cell–associated neurotoxicity syndrome (ICANS), which were graded by American Society for Transplantation and Cellular Therapy (ASTCT) criteria (27). CRS and ICANS were considered AEs of special interest, and infections, cytopenias, injection site reactions and secondary malignancies were considered AEs of clinical interest. Other secondary endpoints included PK, pre-and post-dose serum cytokine levels and incidence of anti-drug antibodies (ADAs) and neutralizing antibodies against elranatamab. Antimyeloma activity was assessed by ORR, time to response (TTR), duration of response (DOR), progression-free survival (PFS) and OS, all based on IMWG criteria for response and assessed by investigator; minimal residual disease (MRD) negativity was assessed by next-generation sequencing at a sensitivity of 1 × 10^−5^ based on IMWG criteria (28).

#### MagnetisMM-3

The primary outcome in MagnetisMM-3 was confirmed ORR assessed by blinded independent committee review (BICR) per IMWG criteria.

Key secondary endpoints for MagnetisMM-3 included DOR, CR rate, PFS and TTR by BICR per IMWG criteria; MRD negativity rate (central laboratory) as assessed by next-generation sequencing at a sensitivity of 1 × 10^−5^ per IMWG criteria; and OS. Safety-related secondary endpoints included AEs (as graded by NCI CTCAE v5.0), AEs of special interest (CRS, ICANS [per ASTCT]) and other AEs of clinical interest (infections, cytopenias, injection site reactions and secondary malignancies).

### Statistical analysis

For MagnetisMM-2, sample size was not based on any statistical consideration. The safety analysis set included all enrolled patients who received ≥1 dose of elranatamab and was used for all analyses unless otherwise stated. The per-protocol analysis set, used for the primary endpoint, included all enrolled patients who received ≥1 dose of elranatamab and either experienced a DLT or did not have major treatment deviations during the DLT observation period. Additional information on analysis sets is provided in the Supplementary Methods. Data were descriptive only, and no statistical tests were conducted.

For MagnetisMM-3, sample size calculations for the full trial population were previously reported (16). For the subset of Japanese patients, sample size was not prespecified, and data are descriptive only. Point estimates of ORR by BICR (primary endpoint) were calculated along with the two-sided exact 95% CIs using the Clopper–Pearson method. The safety analysis set included all enrolled patients who received ≥1 dose of elranatamab and was used for all analyses (including efficacy analysis) unless otherwise stated.

## Results

### Patient disposition

Four patients were screened for eligibility and enrolled in MagnetisMM-2, and 12 Japanese patients were enrolled in MagnetisMM-3.

In MagnetisMM-2, all patients (*N* = 4) received ≥1 dose of elranatamab, were included in the safety analysis set and were part of the per-protocol analysis set for the primary outcome. Three patients (75.0%) discontinued treatment (2 [50.0%] due to progressive disease [PD] and 1 [25.0%] died), and treatment was ongoing in 1 patient (25.0%).

In MagnetisMM-3, all patients (*n* = 12) received ≥1 dose of elranatamab and were included in the safety analysis set. Six patients (50.0%) discontinued treatment (1 [8.3%] due to AEs [thrombocytopenia and neutropenia], 1 [8.3%] due to lack of efficacy and 4 [33.3%] due to PD), and treatment was ongoing in 6 patients (50.0%).

### Baseline characteristics

#### MagnetisMM-2

In MagnetisMM-2, the median age was 68.5 years (range, 49–70) and 3 patients (75.0%) were male (Table 1). All patients had ECOG PS 0–1. One patient (25.0%) had high cytogenetic risk (defined as a t[4;14], t[14;16], or del [17p] chromosomal abnormality). The median time since initial diagnosis was 93.3 months (range, 44–126), and median time since disease progression (to the last prior treatment) to the first elranatamab dose was 6.2 months (range, 1–9).

**Table 1:** Japanese patient demographic and clinical characteristics at baseline in MagnetisMM-2 and MagnetisMM-3

Characteristic	MagnetisMM-2 (*N* = 4)	MagnetisMM-3 (*n* = 12)
Age, median (range), years	68.5 (49–70)	66.5 (47–83)
Male, *n* (%)	3 (75.0)	7 (58.3)
ECOG PS, *n* (%)		
0	2 (50.0)	6 (50.0)
1	2 (50.0)	6 (50.0)
R-ISS stage, *n* (%)		
I	1 (25.0)	3 (25.0)
II	2 (50.0)	8 (66.7)
II	1 (25.0)	1 (8.3)
Cytogenetic risk, *n* (%)		
Standard	3 (75.0)	7 (58.3)
High^a^	1 (25.0)	5 (41.7)
Extramedullary disease, *n* (%)^b^		
Yes	2 (50.0)	1 (8.3)
No	2 (50.0)	11 (91.7)
Type of myeloma, *n* (%)		
IgG	2 (50.0)	8 (66.7)
IgA	0	2 (16.7)
Light chain only	2 (50.0)	2 (16.7)
Type of measurable disease at baseline, *n* (%)		
Serum M-protein	3 (75.0)	8 (66.7)
Urine M-protein	3 (75.0)	4 (33.3)
Serum-free light chain	0	2 (16.7)^c^
Bone marrow plasma cells		
<50%, *n* (%)	4 (100)^d^	8 (66.7)
≥50%, *n* (%)	0	4 (33.3)

ECOG PS, Eastern Cooperative Oncology Group performance status; R-ISS, Revised International Staging System.

^a^Defined as any of the following chromosomal abnormalities: *t* (4,14), *t* (14,16) or del(17p).

^b^Defined as the presence of any plasmacytoma (extramedullary and/or paramedullary with a soft tissue component). By investigator in MagnetisMM-2 and blinded independent committee review in MagnetisMM-3.

^c^Includes patients measurable by free light chain only.

^d^Bone marrow plasma cells were <5% for all participants in MagnetisMM-2.

All patients were triple-class refractory (Table 2). In total, all patients were penta-drug exposed and 1 patient (25.0%) was penta-drug refractory. The median number of prior treatments was 7.5 (range, 6–9); prior BCMA-targeted therapy was received by 1 patient (25.0%; belantamab mafodotin). One patient (25.0%) had received a prior stem cell transplant.

**Table 2:** Prior anti–multiple myeloma therapy among Japanese patients in MagnetisMM-2 and MagnetisMM-3

Anti–multiple myeloma therapy	MagnetisMM-2 (*N* = 4)	MagnetisMM-3 (*n* = 12)
Number of prior therapies, median (range)	7.5 (6–9)	6.0 (2–11)
Prior IMiD, *n* (%)	4 (100)	12 (100)
Lenalidomide	4 (100)	11 (91.7)
Pomalidomide	3 (75.0)	9 (75.0)
Iberdomide	1 (25.0)	0
Thalidomide	0	3 (25.0)
Prior PI, *n* (%)	4 (100)	12 (100)
Bortezomib	4 (100)	12 (100)
Carfilzomib	4 (100)	8 (66.7)
Ixazomib	2 (50.0)	5 (41.7)
Prior anti-CD38 mAb, *n* (%)	4 (100)	12 (100)
Daratumumab	4 (100)	11 (91.7)
Isatuximab	2 (50.0)	6 (50.0)
Triple-class exposed, *n* (%)^a^	4 (100)	12 (100)
Triple-class refractory, *n* (%)^a^	4 (100)	12 (100)
Penta-drug exposed, *n* (%)^b^	4 (100)	7 (58.3)
Penta-drug refractory, *n* (%)^b^	1 (25.0)	7 (58.3)
Prior stem cell transplant, *n* (%)	1 (25.0)	8 (66.7)
Autologous	1 (25.0)	8 (66.7)
Prior BCMA targeted therapy, *n* (%)	1 (25.0)	0
ADC	1 (25.0)	0

^a^Triple-class refers to ≥1 IMiD, 1 PI and 1 anti-CD38 mAb.

^b^Penta-drug refers to ≥2 IMiDs, 2 PIs and 1 anti-CD38 mAb.

ADC, antibody drug conjugate; BCMA, B cell maturation antigen; IMiD, immunomodulatory drug; mAb, monoclonal antibody; PI, proteasome inhibitor.

#### MagnetisMM-3

In MagnetisMM-3, the median age was 66.5 years (range, 47–83) and 7 patients (58.3%) were male (Table 1). All patients had ECOG PS 0–1. Five patients (41.7%) had high cytogenetic risk. The median time since initial diagnosis was 75.1 months (range, 20–176), and median time since disease progression (to the last prior treatment) to the first elranatamab dose was 1.3 months (range, 0.6–5.6).

All patients were triple-class refractory (Table 2). Overall, 7 patients (58.3%) were penta-drug refractory. The median number of prior treatments was 6.0 (range, 2–11), with no patient having received a prior BCMA-targeted therapy and 8 patients (66.7%) having received a prior stem cell transplant.

### Duration of follow-up and treatment

In MagnetisMM-2 and MagnetisMM-3, the median duration of follow up was 12.3 months (range, 7.9–13.1) and 11.1 months (range, 2.4–13.0), respectively. The median duration of treatment was 9.7 months (range, 7.4–13.1) and 10.7 months (range, 0.3–12.9), and the median overall relative dose was 98.2% (range, 88.7–100.1) and 73.9% (range, 47.2–100.0) in MagnetisMM-2 and MagnetisMM-3, respectively.

### Safety

#### MagnetisMM-2

No DLTs were reported in any patients in MagnetisMM-2 (primary endpoint). All 4 patients (100%) experienced TEAEs (Table 3). Three patients (75.0%) had grade 3/4 TEAEs. The most commonly occurring TEAEs of any grade were neutropenia and pneumonia (3 patients [75.0%] each) (Table 4). One grade 5 TEAE was reported: the patient had sudden death due to an unknown cause 13 days after the last dose of elranatamab. This death was considered treatment related.

**Table 3:** All-causality treatment-emergent adverse events among Japanese patients in MagnetisMM-2 and MagnetisMM-3

	MagnetisMM-2 (*N* = 4)	MagnetisMM-3 (*n* = 12)
Patients with TEAEs, *n* (%)	4 (100)	12 (100)
Patients with TEAEs of maximum grade 3 or 4, *n* (%)	3 (75.0)	12 (100)
Patients with TEAEs of maximum grade 5, *n* (%)	1 (25.0)	0
Patients with TEAEs leading to permanent treatment discontinuation, *n* (%)	0	1 (8.3)
Patients with TEAEs leading to dose interruption or dose reduction, *n* (%)	3 (75.0)	11 (91.7)
Dose interruption	3 (75.0)	10 (83.3)
Dose reduction	1 (25.0)	6 (50.0)

TEAE, treatment-emergent adverse event.

**Table 4:** All-causality treatment-emergent adverse events occurring in ≥2 Japanese patients in either MagnetisMM-2 or MagnetismMM-3

Adverse event, *n* (%)	MagnetisMM-2 (*N* = 4)	MagnetisMM-3 (*n* = 12)
Haematologic AEs^a^		
Neutropenia	3 (75.0)	10 (83.3)
Lymphopenia	2 (50.0)	3 (25.0)
Leukopenia	1 (25.0)	5 (41.7)
Anaemia	1 (25.0)	2 (16.7)
Thrombocytopenia	0	6 (50.0)
Non-haematologic AEs		
Cytokine release syndrome	4 (100.0)	7 (58.3)
Injection site reaction^b^	2 (50.0)	7 (58.3)
Hypogammaglobulinemia	2 (50.0)	3 (25.0)
Pyrexia	2 (50.0)	3 (25.0)
Pneumonia	3 (75.0)	0
Diarrhoea	2 (50.0)	1 (8.3)
Cytomegalovirus infection	1 (25.0)	2 (16.7)
Vomiting	0	3 (25.0)
Upper respiratory tract infection	0	3 (25.0)
Decreased weight	2 (50.0)	0
Palpitations	0	2 (16.7)
Adrenal insufficiency	0	2 (16.7)
Conjunctival haemorrhage	0	2 (16.7)
Large intestine polyp	0	2 (16.7)
Malaise	1 (25.0)	2 (16.7)
Fatigue	0	2 (16.7)
Injection site erythema	0	2 (16.7)
Injection site pruritus	0	2 (16.7)
Pneumonia bacterial	0	2 (16.7)
Decreased appetite	0	2 (16.7)
Headache	1 (25.0)	2 (16.7)
Dizziness	0	2 (16.7)
Pruritus	0	2 (16.7)

^a^See Supplementary Methods for details on clustered terms for cytopenias.

^b^Medical Dictionary for Regulatory Activities high-level term.

AE, adverse event.

No patients had TEAEs leading to permanent treatment discontinuation. Three patients (75.0%) had TEAEs leading to dose interruption (hypogammaglobulinemia, pneumonia and malaise), and 1 patient (25.0%) had a TEAE leading to dose reduction (pneumonia); all were considered related to treatment.

CRS occurred in all 4 patients (100%; Table 5), with grade 1 occurring in 2 patients and grades 2 and 3 CRS in 1 patient each. All CRS events occurred after the first dose with no events after the second or later doses. The median time to onset and resolution of CRS after the first dose was 2.0 days (range, 2.0–2.0) and 2.5 days (range, 2.0–4.0), respectively. Overall, 3 patients (75.0%) received tocilizumab and/or steroids for CRS (3 received tocilizumab and 1 received steroids). No ICANS was reported in the study. Infections were reported in 3 patients (75.0%) with grade 3/4 occurring in 2 (50.0%). The most frequently reported all-causality infection was pneumonia (3 patients [75.0%]). All other infections (bronchitis, conjunctivitis, cytomegalovirus infection, cytomegalovirus infection reactivation, nasopharyngitis, oral candidiasis and otitis media) were reported in individual patients. Cytopenia was reported in all 4 patients (100%), all at grade 3/4. Injection site reactions were observed in 2 patients (50.0%), and none were grade 3/4. All 4 patients (100%) received anti-viral prophylaxis. Two patients (50.0%) received anti-*Pneumonocystis jirovecii* prophylaxis. Anti-fungal prophylaxis was administered to 2 patients (50.0%), while anti-bacterial prophylaxis was administered to 3 patients (75.0%) (Supplementary Table 1).

**Table 5:** Adverse events of interest (all causalities) among Japanese patients in MagnetisMM-2 and MagnetisMM-3

Adverse event, *n* (%)	MagnetisMM-2 (*N* = 4)		MagnetisMM-3 (*n* = 12)	
	All grades	Grade 3/4	All grades	Grade 3/4
Cytokine release syndrome	4 (100)	1 (25.0)	7 (58.3)	0
ICANS	0	0	0	0
Infections	3 (75.0)	2 (50.0)	7 (58.3)	2 (16.7)
Cytopenia	4 (100)	4 (100)	11 (91.7)	11 (91.7)
Injection site reaction	2 (50.0)	0	7 (58.3)	0

ICANS, immune effector cell–associated neurotoxicity syndrome.

#### MagnetisMM-3

All 12 patients (100%) in MagnetisMM-3 experienced grade 3/4 TEAEs, which were all grade 3/4 in nature (Table 3). The most commonly occurring TEAEs of any grade were neutropenia (10 patients [83.3%]) and thrombocytopenia (6 patients [50.0%]; Table 4). There were no grade 5 TEAEs. Ten patients (83.3%) received granulocyte colony-stimulating factor.

Eleven patients (91.7%) had TEAEs leading to dose interruption or reduction; the most common were neutropenia (9 [75.0%]), leukopenia (2 [16.7%]), bacterial pneumonia (2 [16.7%]) and upper respiratory tract infection (2 [16.7%]). One patient (8.3%) had dose interruption or reduction due to CRS.

CRS occurred in 7 patients (58.3%; Table 5), with grade 1 occurring in 5 patients and grade 2 in 2 patients. All 7 patients had a CRS event after the first step-up dose, and 3 (25.0%) had CRS after the second step-up dose. The median time to onset of CRS after the first and second doses was 2.0 days (range, 2.0–4.0) and 2.0 days (range, 2.0–3.0), respectively. The median time to resolution of CRS was 2.0 days (range, 1.0–3.0) for the first dose and 2.0 days (range, 1.0–19.0) for the second dose. Overall, 3 (42.9%) and 2 (66.7%) patients received tocilizumab and/or steroids for CRS after the first (*n* = 3 tocilizumab, *n* = 2 steroids) and second (*n* = 2 tocilizumab, 0 received steroids) step-up dose, respectively. No patients had CRS after later doses. No ICANS was reported. Infections were reported in 7 patients (58.3%), with grade 3/4 occurring in 2 patients (16.7%). The most frequently reported all-causality infection was upper respiratory tract infection (3 patients [25.0%]). Cytomegalovirus infection and bacterial pneumonia were reported in 2 patients (16.7%) each, and bacteraemia and *Pneumocystis jirovecii* pneumonia were reported in 1 (8.3%) patient each. Cytopenia was reported in 11 patients (91.7%), all at grade 3/4. Seven patients (58.3%) experienced injection site reactions, none of which were grade 3/4 reactions. All 12 patients (100%) received anti-viral prophylaxis. Ten patients (83.3%) received anti-*P. jirovecii* prophylaxis. Anti-fungal prophylaxis was administered to 2 patients (16.7%), while anti-bacterial prophylaxis was administered to 1 patient (8.3%) (Supplementary Table 1).

### Pharmacokinetics, immunogenicity and cytokine profiles

Robust data for PK, immunogenicity and cytokine profiles for Japanese patients were available only from MagnetisMM-2.

A descriptive summary of total serum elranatamab PK parameters following administration of a single priming dose of 600 μg/kg is shown in Table 6. Elranatamab exposure increased in a sustained manner over time through Cycle 4 Day 1 and generally remained consistent thereafter (Supplementary Fig. 1). Two patients (50.0%) were positive for ADAs to elranatamab; neither were positive for neutralizing antibodies to elranatamab. ADAs observed in these 2 patients were detected at a single point (Day 260 and Day 386, respectively). One patient who developed ADAs at Day 260 showed very good partial response at that time and later achieved CR with continued elranatamab treatment. The other patient who developed ADAs at Day 386 had previously achieved CR at Day 352, but discontinued elranatamab treatment due to disease progression at Day 386. In general, serum cytokine increases were observed predominantly following the first dose and peaked between 8 and 72 hours post-dose, corresponding with CRS incidence predominantly following the first dose. Representative cytokine profile data are shown for interleukin 2, interleukin 6, interferon γ and tumor necrosis factor in Supplementary Fig. 2.

**Table 6:** Descriptive summary of serum total elranatamab pharmacokinetic parameters following administration of a single priming dose of 600 μg/kg

Parameter, Units^a^	MagnetisMM-2 (*N* = 4)	MagnetisMM-1 (*N* = 14)^15,b^
AUC_tau_, μg·day/ml	21.16 (42)	17.70 (37)
AUC_tau_ (dn), μg·day/ml/mg	0.5146 (41)	0.4213 (47)
*C*_max_, μg/ml	4.382 (33)	4.084 (37)
*C*_max_ (dn), μg/ml/mg	0.1064 (35)	0.0973 (46)
*T*_max_, median (range), days	7.00 (2.96–8.00)	6.98 (6.93–6.99) ^c^

*N*, number of participants contributing to the summary statistics.

^a^Geometric mean (geometric % coefficient of variation) unless otherwise indicated.

^b^MagnetisMM-1 data shown for participants in Part 1.1 (overall patient population) who received a priming dose of 600 μg/kg, followed by 1000 μg/kg full dose.

^c^Interquartile range shown for MagnetisMM-1 data.

AUC_tau_, area under the plasma concentration-time curve from time zero to the end of the dosing interval; *C*_max_, maximum observed plasma concentration; dn, dose normalization; *T*_max_, time of the maximum observed plasma concentration.

### Efficacy

#### MagnetisMM-2 (investigator assessed)

**Figure 1:**
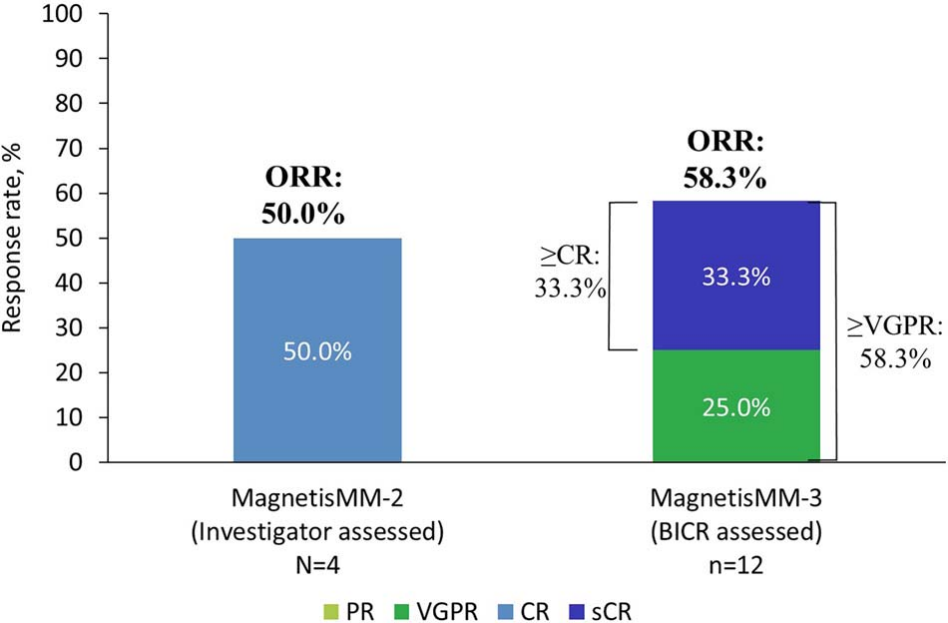
Best overall response to elranatamab in Japanese patients in MagnetisMM-2 and MagnetisMM-3. BICR, blind independent central review; CR, complete response; ORR, objective response rate; PR, partial response; sCR, stringent complete response; VGPR, very good partial response.

**Figure 2:**
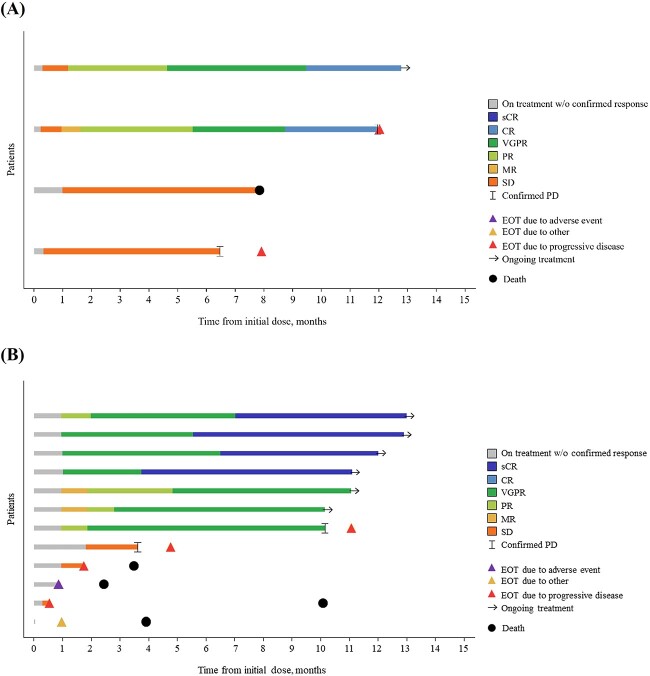
Swimmer’s plots of duration of response, progression-free survival and overall survival for Japanese patients in MagnetisMM-2 and MagnetisMM-3. (A) Swimmer’s plot of the four patients in MagnetisMM-2 (investigator assessed and based on IMWG criteria for response). (B) Swimmer’s plot of the 12 patients in MagnetisMM-3 (BICR assessed and based on IMWG response criteria for MM). The end of the bar is either a PFS event or the last valid assessment if no PFS event. CR, complete response; EOT, end of treatment; IMWG, International Myeloma Working Group; MR, minimal response; PD, progressive disease; PR, partial response; sCR, stringent complete response; SD, stable disease; VGPR, very good partial response.

At the data cutoff, the ORR was 50.0% (95% CI, 6.8–93.2 [*n* = 2]; Fig. 1). The best overall response was CR in 2 patients (Fig. 2A), stable disease in 1 patient (25.0%) and PD in 1 patient (25.0%). The one patient who had prior BCMA-targeted therapy (ADC, belantamab mafodotin) achieved CR. For the 2 patients who responded, median (range) TTR was 0.8 months (0.3–1.3); the median (range) time to CR or better was 8.2 months (7.8–8.5). The median DOR could not be estimated by Kaplan–Meier estimates of time to event, but the DOR was between 10.7 months and >12.5 months (CR still ongoing in 1 patient at data cutoff); the probability of maintaining response at 6 months and 12 months was 100.0% and 50%, respectively. MRD negativity at a sensitivity threshold of 1 × 10^−5^ was achieved in 100% of patients with CR or better and who were evaluable for MRD (*n* = 1), corresponding to 50.0% of patients with CR or better.

Median PFS and OS for patients in the Japanese subpopulation were not assessed by Kaplan–Meier estimates of time to event. PFS was >6 months for all 4 patients (range, 6.5–12.8); 2 patients (50.0%) experienced disease progression, 1 patient (25.0%) died and treatment was ongoing in 1 patient (25.0%). OS was >11 months in the 3 patients (75.0%) who continued OS follow up (range, 11.8–13.1).

#### MagnetisMM-3 (BICR assessed)

At the data cutoff, the ORR was 58.3% (95% CI, 27.7–84.8 [*n* = 7]; Fig. 1). Four patients (33.3%) achieved stringent CR, and 3 patients (25.0%) achieved very good partial response (Fig. 2B). At data cutoff, 2 responders (16.7%) were still on treatment without disease progression and with confirmed CR-2. Stable disease was observed in 4 patients (33.3%). In patients who responded, the median TTR was 0.99 months (range, 0.95–1.87); the median time to CR or better was 6.03 months (range, 3.75–7.03). The median DOR could not be assessed by Kaplan–Meier estimates of time to event, but the DOR was >9 months for all responders; the probability of maintaining response at 6 and 12 months was 100.0% and 83.3%, respectively. MRD negativity at a sensitivity threshold of 1 × 10^−5^ was achieved in 100% of patients with CR or better and who were evaluable for MRD (*n* = 3), corresponding to 75.0% of patients with CR or better.

Median PFS and OS for patients in the Japanese subpopulation were not assessed by Kaplan–Meier estimates of time to event. The probability of PFS at 12 months was 75.0% (95% CI, 31.5–93.1); 2 patients (16.7%) experienced disease progression. The probability of being alive at 12 months was 66.7% (95% CI, 33.7–86.0); 4 patients (33.3%) died, all due to disease progression.

## Discussion

Overall, the results presented here confirm and extend previous reports from the MagnetisMM-1 study and the overall population from the MagnetisMM-3 study (15,16). MagnetisMM-2 showed that elranatamab PK was comparable between Japanese patients and patients in the trial population in MagnetisMM-1. No new safety signals were identified for elranatamab in Japanese patients, compared with the overall MagnetisMM-1 and MagnetisMM-3 trial populations. One case of sudden death was reported in MagnetisMM-2 and was the only case observed to date in 4 MagnetisMM trials (29).

Elranatamab showed a manageable safety profile among Japanese patients, with no DLTs reported in MagnetisMM-2. All patients had CRS in MagnetisMM-2, and 1 patient developed grade 3 CRS the day after the 600-μg/kg priming dose. SC elranatamab with step-up doses of 12 mg on Day 1 and 32 mg on Day 4 followed by full treatment of 76 mg weekly (starting on Day 8) has recently been recommended for the overall patient population receiving elranatamab (30). In MagnetisMM-3, in which all Japanese patients received this dosing schedule, the incidence of CRS was lower (58.3%) than in MagnetisMM-2, and there were no grade ≥3 events, supporting this alternate priming dose regimen for Japanese patients. The incidence and severity of CRS among Japanese patients in MagnetisMM-3 were comparable to the 56.3% (with no grade ≥3 events) reported for the overall trial population and consistent with previous reports showing a trend for reduced CRS incidence and severity for bispecific antibodies compared with chimeric antigen receptor T-cell therapies for RRMM (16,31–33). Most patients in both studies experienced infection, consistent with prior reports indicating an increased risk of infection among patients with MM receiving BCMA-directed immunotherapies (34–38). Therefore, it is important to closely monitor and recognize infection early. Recently published consensus guidelines from the IMWG provide recommendations on how to promptly manage infections and emphasize the importance of antimicrobial prophylaxis and immunoglobulin replacement therapy for patients with MM receiving BCMA-targeted T-cell–redirecting therapies (39). There were no instances of ICANS in the Japanese patients. A similar low incidence of neurotoxic effects was reported for elranatamab in the BCMA-naive MagnetisMM-3 population, where ICANS (all grade 1 or 2) occurred in 3.4% of patients, and for other BCMA-targeting bispecific antibodies such as teclistamab, where ICANS incidence was 3.0% (all grade 1 or 2) (31,40).

Elranatamab PK for Japanese patients was similar to those of other population groups. Maximum serum elranatamab concentrations of ~4.4 μg/ml were reached ~7 days after a single 600 μg/kg priming dose in MagnetisMM-2, similar to the maximum serum elranatamab concentrations of ~4.1 μg/ml reached ~7 days after the 600 μg/kg priming dose in MagnetisMM-1 (15). Cytokine increases occurred predominantly after the first dose, consistent with the increased incidence of CRS following the first dose. Half (2 of 4) of the patients in MagnetisMM-2 developed ADAs at a single point in time, late in treatment (Day 260 and Day 386), but neither had neutralizing ADAs. One patient continued elranatamab treatment and achieved CR. The incidence of ADAs is higher than the 8.6% reported for MagnetisMM-1; however, this is likely to have been influenced by the small sample size (*N* = 4) (15). Elranatamab demonstrated efficacy in Japanese patients, with ORRs among Japanese patients of 50.0% (MagnetisMM-2) and 58.3% (MagnetisMM-3), which were similar to that of the MagnetisMM-3 overall trial population (61.0%) (16). CR was observed in one-third of patients. In MagnetisMM-2, half of patients achieved CR or better, including the patient who had received prior BCMA antibody–drug conjugate.

The key limitation of MagnetisMM-2 is the small patient population. To improve the generalizability of these results, we also included in this analysis data from Japanese patients in the MagnetisMM-3 trial. Another limitation is the short follow-up time, which limits an assessment of response deepening over time.

In conclusion, SC elranatamab with step-up doses of 12 mg on Day 1 and 32 mg on Day 4 followed by a full dose of 76 mg weekly (starting on Day 8) showed a favourable benefit/risk profile in Japanese patients with RRMM, extending previous reports in the overall patient population. Efficacy was observed in Japanese patients with triple-class refractory RRMM, without any additional safety signals observed in MagnetisMM-2 or MagnetisMM-3. These results broaden the population that can benefit from this promising new therapy and support further studies to develop elranatamab.

## Supplementary Material

Supplementary_figure_1_hyae068

Supplementary_figure_2_hyae068

Supplementary_materials_hyae068

Supplementary_table_1_hyae068

## Data sharing statement

Upon request, and subject to review, Pfizer will provide the data that support the findings of this study. Subject to certain criteria, conditions and exceptions, Pfizer may also provide access to the related individual de-identified participant data. See https://www.pfizer.com/science/clinical-trials/trial-data-and-results for more information.
