# Supplementary figures and images for: Elranatamab in Japanese patients with relapsed/refractory multiple myeloma: results from MagnetisMM-2 and MagnetisMM-3
Source: Jpn J Clin Oncol. 2024 May 24;54(9):991–1000. doi: 10.1093/jjco/hyae068 (PMC11374885; doi:10.1093/jjco/hyae068)

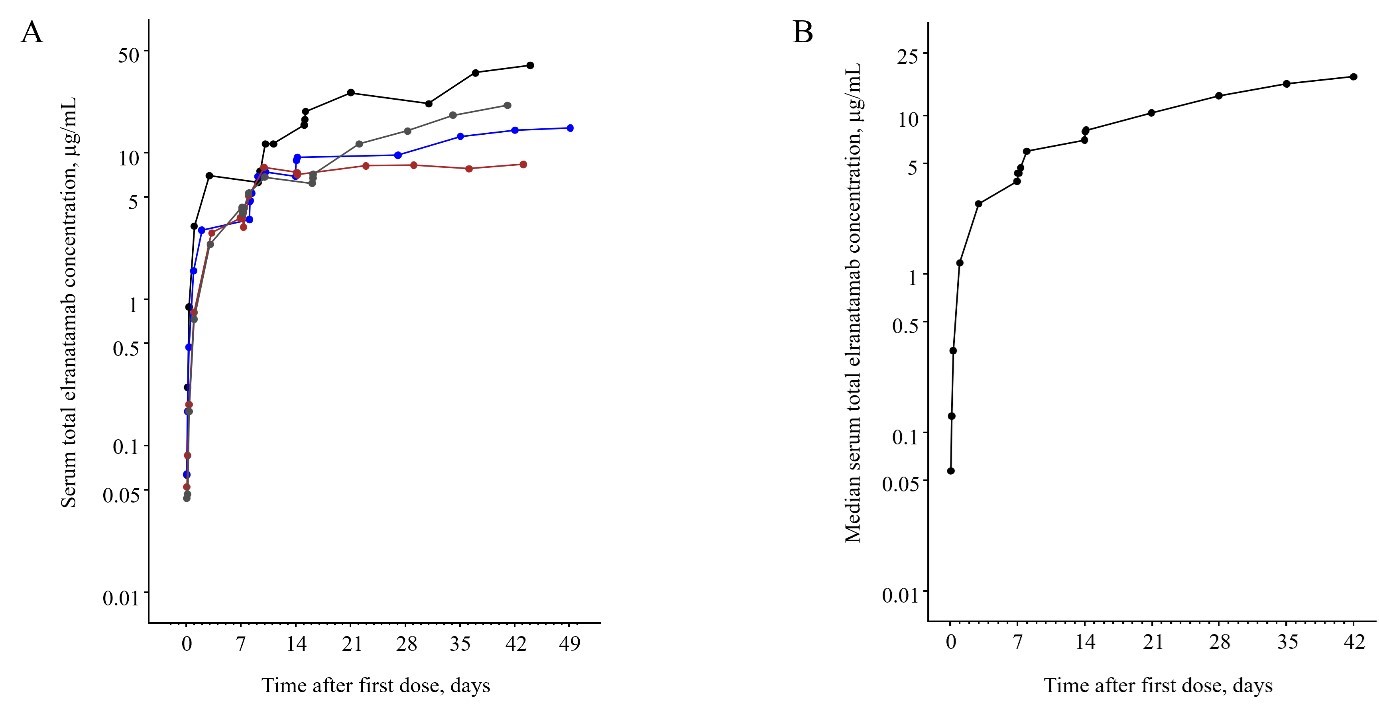

Supplement: Supplementary_figure_1_hyae068 [file supplementary_figure_1_hyae068.jpeg]

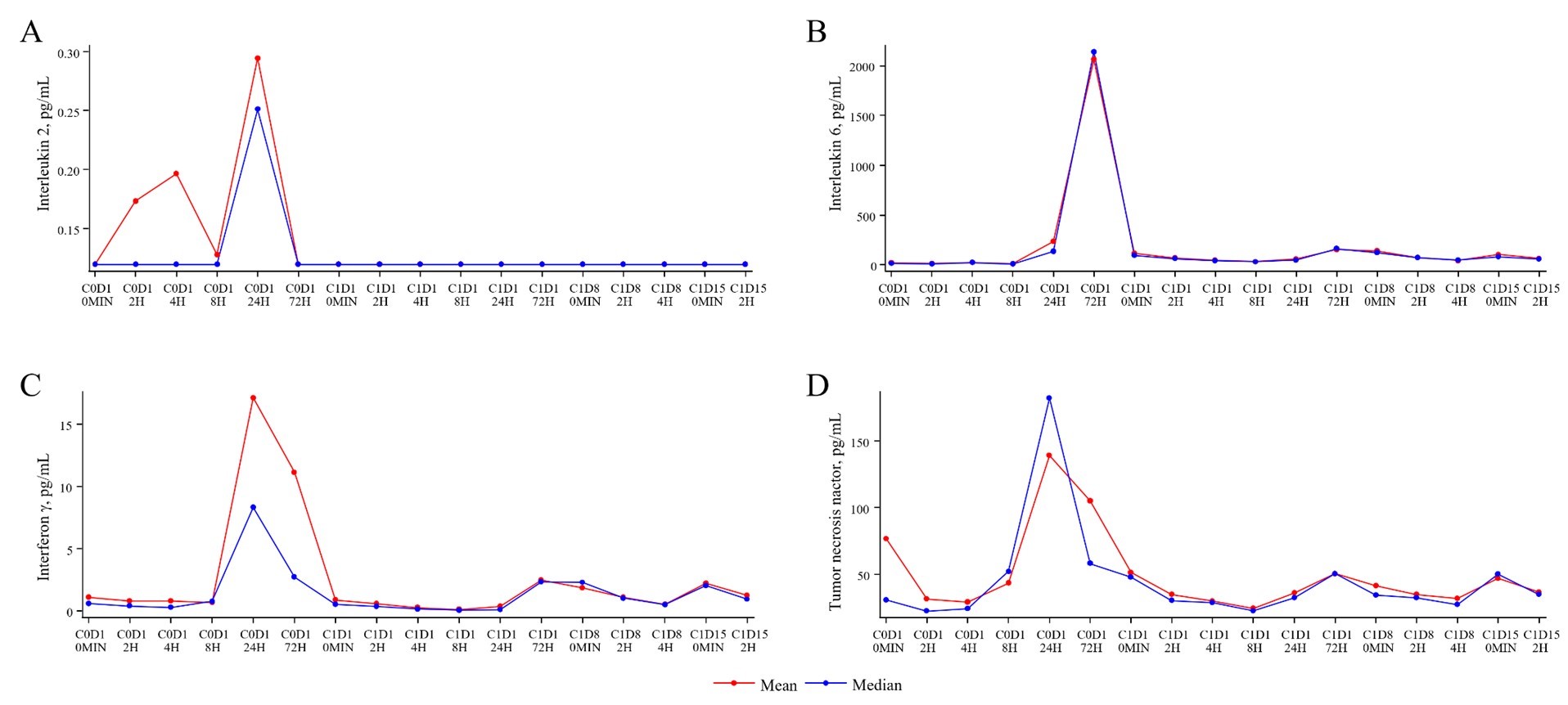

Supplement: Supplementary_figure_2_hyae068 [file supplementary_figure_2_hyae068.jpeg]
