# Supplementary material for: Elranatamab in Japanese patients with relapsed/refractory multiple myeloma: results from MagnetisMM-2 and MagnetisMM-3
Source: Jpn J Clin Oncol. 2024 May 24;54(9):991–1000. doi: 10.1093/jjco/hyae068 (PMC11374885; doi:10.1093/jjco/hyae068)
Supplement: Supplementary_materials_hyae068 [file supplementary_materials_hyae068.docx]

# **Supplementary Methods**

***Description of inclusion criteria parameters in both MagnetisMM-2 and MagnetisMM-3***

1) Measurable disease was defined by ≥1 of the following:

- Serum M-protein ≥0.5 g/dL (5 g/L)
- Urine M-protein ≥200 mg/24 hours
- Serum free light chain >100 mg/L (in MagnetisMM-2) or ≥100 mg/L (in MagnetisMM-3), and an abnormal κ:λ ratio

2) Adequate bone marrow function defined as:

- ANC ≥1.0 × 10^9^/L
- Platelets ≥25 × 10^9^/L
- Hemoglobin ≥8.0 g/dL

3) Adequate renal function defined as creatine clearance ≥30 mL/min

4) Adequate hepatic function defined as:

- Total bilirubin ≤2.0 mg/dL (MagnetisMM-2) except in patients with Gilberts Syndrome (must have a total bilirubin <3.0 mg/dL) or ≤2 × upper limit of normal (ULN; ≤3 × ULN if documented Gilbert syndrome) (MagnetisMM-3)
- Aspartate aminotransferase ≤2.5 × ULN
- Alanine aminotransferase ≤2.5 × ULN

***Additional inclusion criteria for patients in both MagnetisMM-2 and MagnetisMM-3***

- AEs associated with any prior therapies must be resolved to baseline severity or Common Terminology Criteria for Adverse Events (CTCAE) grade ≤1 or not constituting a safety risk per investigator judgment
- Patients are capable of giving signed informed consent, including compliance with the requirements and restrictions listed in the informed consent document and respective study protocols
- Patients are willing and able to comply with all scheduled visits, treatment plan, laboratory tests, lifestyle considerations, and other study procedures
- Female patients are eligible to participate if they are not pregnant or breastfeeding and ≥1 of the following conditions applies:
  - The patient is not a woman of childbearing potential ***or***
  - The patient is a woman of childbearing potential and using a contraceptive method that is highly effective (failure rate of <1% per year) during the intervention period for ≥90 days after the last dose of study intervention

***Additional inclusion criteria for patients in MagnetisMM-2 only***

- ECOG PS of 3 was permitted if due solely to bone pain

***Additional inclusion criteria for patients in MagnetisMM-3 only***

- Relapsed or refractory (defined as having disease progression while on therapy or within 60 days of last dose in any line, regardless of response) to the last anti-MM therapy
- Left ventricular ejection fraction ≥40% as determined by a MUGA scan or ECHO

***Key exclusion criteria for patients in both MagnetisMM-2 and MagnetisMM-3***

- POEMS syndrome
- Any other active malignancy with 3 years prior to enrollment, except for adequately treated basal cell or squamous cell skin cancer, or carcinoma in situ
- Ongoing grade ≥2 peripheral sensory or motor neuropathy
- History of Guillain-Barré syndrome (GBS) or GBS variant, history of grade ≥3 peripheral motor polyneuropathy, or history of any-grade peripheral sensory or motor neuropathy with prior BCMA-directed therapy
- Stem cell transplant within 12 weeks prior to enrollment or active graft versus host disease
- Active, uncontrolled bacterial, fungal, or viral infection, including hepatitis B virus, hepatitis C virus, known HIV and SARS-CoV2
- Known or suspected hypersensitivity to component of elranatamab
- Other medical or psychiatric condition including recent (within the past year) or active suicidal ideation/behavior or laboratory abnormality that may increase the risk of study participation
- Live attenuated vaccine within 4 weeks of the first dose of study intervention

***Key exclusion criteria for patients in MagnetisMM-2 only***

- Major surgery within 4 weeks prior to study entry
- Requirement for systemic immunosuppressive medication (eg, >10 mg of prednisone or equivalent)
- Refractoriness to platelet or red blood cell transfusions
- Current requirement for chronic blood product support
- Clinically relevant abnormalities as determined by baseline 12-lead electrocardiogram
- Any of the following in the previous 6 months: myocardial infarction; long QT syndrome; Torsade de Pointes; arrhythmia; serious conduction system abnormalities; unstable angina; coronary/peripheral artery bypass graft; symptomatic congestive heart failure, New York Heart Association class III or IV; cerebrovascular accident; transient ischemic attack; symptomatic pulmonary embolism; and/or other clinical significant episode of thromboembolic disease or ongoing cardiac dysrhythmias of CTCAE grade ≥2 or atrial fibrillation of any grade
- Hypertension that cannot be controlled by medications (eg, >150/100 mmHg)
- Participation in other studies involving investigational drug(s) within 4 weeks prior to study entry; patients may be included if 5 times the elimination half-life of the drug has passed
- Donor lymphocyte infusion within 30 days prior to study entry
- Radiation therapy within 2 weeks prior to study entry
- History of active immune disorders and other conditions that compromise or impair the immune system
- Any form of primary immunodeficiency
- History of CTCAE grade ≥3 immune-mediated AE considered related to prior immunomodulatory therapy

***Key exclusion criteria for patients in MagnetisMM-3 only***

- Smoldering multiple myeloma
- Active plasma cell leukemia
- Amyloidosis
- Major surgery within 14 days prior to study entry
- Impaired cardiovascular function or clinically significant cardiovascular diseases, defined as any of the following within the previous 6 months: acute myocardial infarction or acute coronary syndromes, clinically significant cardiac arrythmias, thromboembolic or cerebrovascular events, or prolonged QT syndrome (or triplicate average QTcF >470 ms at screening)
- Prior treatment with an anti-BCMA bispecific antibody
- Previous administration with an investigational drug within 30 days or 5 half-lives preceding the first dose of study intervention used in the study (whichever is longer)

***Additional analysis sets in MagnetisMM-2 only***

1. PK parameter analysis population

- All enrolled patients who were treated and did not have protocol deviations influencing PK assessment
- Had sufficient information to estimate ≥1 of the PK parameters of interest

1. PK concentration population

- All enrolled patients who were treated and had ≥1 analyte concentration

1. Biomarker analysis set

- All enrolled patients with ≥1 of the biomarkers evaluated at 1 baseline biomarker assessment

1. Immunogenicity analysis set

- All enrolled patients who received ≥1 dose of study intervention and had ≥1 sample tested for ADAs

***Clustered terms for hematologic adverse events in MagnetisMM-2 and MagnetisMM-3***

- Anemia: anemia, hemoglobin decreased, red blood cell count decreased, hematocrit decreased, normochromic anemia, normocytic anemia, normochromic normocytic anemia
- Leukopenia: leukopenia, white blood cell count decreased
- Lymphopenia: lymphopenia, lymphocyte count decreased, lymphocyte percentage decreased, CD4 lymphocytes decreased, CD4 lymphocyte percentage decreased, CD8 lymphocytes decreased, CD8 lymphocyte percentage decreased
- Neutropenia: neutropenia, neutrophil count decreased, neutrophil percentage decreased, cyclic neutropenia, agranulocytosis, granulocytopenia, granulocyte count decreased
- Thrombocytopenia: thrombocytopenia, platelet count decreased
