# Supplementary material for: Elranatamab in Japanese patients with relapsed/refractory multiple myeloma: results from MagnetisMM-2 and MagnetisMM-3
Source: Jpn J Clin Oncol. 2024 May 24;54(9):991–1000. doi: 10.1093/jjco/hyae068 (PMC11374885; doi:10.1093/jjco/hyae068)
Supplement: Supplementary_table_1_hyae068 [file supplementary_table_1_hyae068.docx]

**Supplementary Table 1.** Antimicrobial agents administered for infection prophylaxis in MagnetisMM-2 and MagnetisMM-3

| **Antimicrobial prophylaxis, n (%)^a^** | **MagnetisMM-2  (N=4)** | **MagnetisMM-3  (n=12)** |
| --- | --- | --- |
| Antiviral | 4 (100) | 12 (100) |
| Aciclovir | 3 (75.0) | 12 (100) |
| Valaciclovir | 1 (25.0) | 0 |
| Valganciclovir hydrochloride | 1 (25.0) | 1 (25.0) |
| Anti-*Pneumocystis jirovecii* | 2 (50.0) | 10 (83.3) |
| Sulfamethoxazole and/or Trimethotrim | 2 (50.0) | 10 (83.3) |
| Anti-fungal | 2 (50.0) | 2 (16.7) |
| Fluconazole | 2 (50.0) | 1 (8.3) |
| Itraconazole | 0 | 1 (8.3) |
| Anti-bacterial | 3 (75) | 1 (8.3) |
| Levofloxacin | 3 (75.0) | 1 (8.3) |
| IVIG therapy | 3 (75.0) | 8 (66.7) |
| Immunoglobulin human normal | 3 (75.0) | 5 (41.7) |
| Immunoglobulin G human | 0 | 2 (16.7) |
| Immunoglobulin human normal; Macrogol | 0 | 2 (16.7) |

^a^ Criteria for distinguishing antimicrobial agent administration for prophylaxis vs other indication (eg, treatment of active infection) included continuous treatment for at least 14 days and not administered to treat an adverse event. IVIG=intravenous immunoglobulin.
